# Physical activity and sleep disturbances in individuals with alcohol use disorder: protocol of the APhysOH pilot study for a randomized controlled trial

**DOI:** 10.1186/s13722-026-00683-1

**Published:** 2026-06-13

**Authors:** Lilou Duquet, Anastasia Boitel, Silvio Galli, Karine Charriere, Frédéric Mauny, Emmanuel Haffen, Julie Giustiniani

**Affiliations:** 1 Université Marie et Louis Pasteur, INSERM, UMR 1322 LINC, Besançon, F-25000 France; 2 CHU Besançon, Service de Psychiatrie de l’Adulte, Besançon, F-25000 France; 3 CHU Besançon, Service exploration du Sommeil, Besançon, F-25000 France; 4 CHU Besançon, uMETh, CIC 1431 Inserm, Besançon, F-25000 France; 5 Université Marie et Louis Pasteur, CNRS, Chrono-environnement (UMR6249), Besançon, F-25000 France; 6 CHU Besançon, CIC 1431 Inserm, Domaine Santé Mentale et Neurosciences, Service de Psychiatrie de l’Adulte, Besançon, F-25000 France

**Keywords:** Addiction, Physical activity, Sleep, Alcohol, Exercise

## Abstract

**Background:**

Physical activity has been shown to improve sleep and may serve as an effective adjunctive therapy in the treatment of alcohol use disorder (AUD). However, few studies have examined the impact of Adapted Physical Activity (APA) programs on sleep in individuals with AUD. This pilot randomized controlled trial (RCT), APhysOH, aims to assess the feasibility of an RCT evaluating the effects of an APA intervention on sleep, alcohol use, and mental and physical health outcomes in individuals with AUD.

**Methods:**

Forty participants will be recruited and randomized to either an intervention group, referred to as the supervised group (*n* = 30), or a control group, referred to as the autonomous group (*n* = 10). The intervention consists of a 12-week APA program tailored to the abilities of patients with AUD and designed to improve sleep. It includes aerobic and resistance-based exercises delivered through three one-hour morning sessions per week. The control group will continue usual care and receive educational material on the benefits of physical activity for AUD and sleep. The primary objective of this study is to assess feasibility. Secondary objectives are to evaluate the impact of a supervised APA program on sleep, addiction severity, physical fitness, functional capacity, and mental health in patients with AUD. Both objective and subjective measures will be used, including biomarkers, actigraphy, physical performance tests, validated questionnaires, and diaries recording sleep, physical activity, and alcohol consumption.

**Discussion:**

Findings from this pilot RCT protocol will inform the design of future large-scale trials by evaluating the feasibility of this type of intervention. The study will also generate preliminary data to estimate the potential effects of APA on sleep, addiction severity, and physical and mental health in individuals with AUD.

**Trial registration:**

ClinicalTrials.gov identifier: NCT06706349. Registered on 2 June 2024; last updated on 26 November 2024. Sponsor: CHU Besançon, France.

**Graphical Abstract:**

**Figure:**
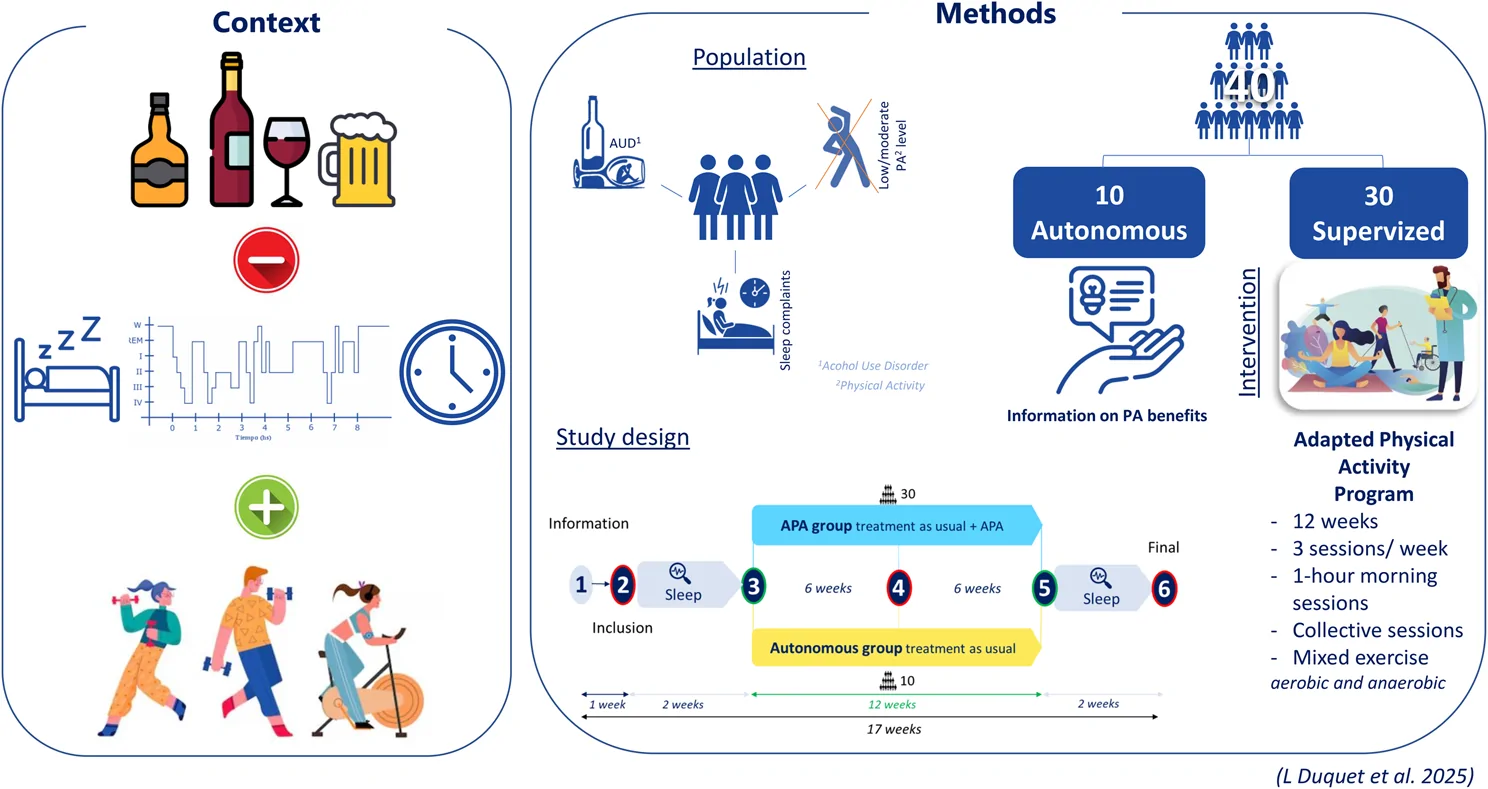

**Supplementary information:**

The online version contains supplementary material available at https://doi.org/10.1186/s13722-026-00683-1.

## Introduction

Worldwide, in 2019, approximately 2.6 million deaths were attributable to alcohol consumption according to the World Health Organization (WHO). The burden of alcohol-attributable mortality is concentrated among individuals with higher levels of consumption, with risk increasing sharply as intake rises [1]. Alcohol use disorder (AUD) is a global public health concern that affects psychological, physical, and social functioning [2]. Patients with AUD are individuals diagnosed with alcohol addiction [3]. In this population, sleep problems, such as insomnia, are overrepresented [4], and 79% of patients with AUD report poor sleep quality [5].

The detrimental effects of alcohol on sleep begin during acute consumption. Sleep onset latency (SOL) is reduced during the declining phase of blood alcohol concentration after alcohol consumption [6]. In some studies, this effect has been linked to the fact that one in ten individuals self-medicates sleep disturbances with alcohol [7]. Acute alcohol consumption induces multiple awakenings, particularly during the second half of the night, leading to reductions in total sleep time (TST) and sleep efficiency (SE) [4].

In cases of chronic alcohol consumption, the detrimental effects of alcohol on sleep are amplified. Chronic alcohol use disrupts sleep–wake cycles [8], increases sleep fragmentation and rapid eye movement (REM) sleep, and reduces slow-wave sleep (SWS) [4, 6], thereby reducing sleep quality. Alcohol use is also associated with circadian misalignment due to melatonin suppression and reduced responsiveness of circadian photoreceptors [9, 10]. During withdrawal, patients with AUD often experience increased SOL, alongside delayed melatonin onset and peak levels. Although partial recovery is possible shortly after withdrawal, some alterations may persist over time, including increased REM sleep, sleep fragmentation, wake after sleep onset (WASO), SOL, stage 1 sleep, and insomnia, along with reduced SWS, SE, and TST [4, 6].

Thus, alcohol impairs sleep, while poor sleep is itself a risk factor for chronic conditions, including addiction. Sleep disturbances are associated with an increased risk of relapse and may contribute to the maintenance and progression of AUD, including during abstinence [4, 6]. Specifically, sleep fragmentation, elevated REM sleep, and periodic limb movements are significant predictors of relapse [6]. Individuals who habitually use alcohol or hypnotic medications as sleep aids before admission to a residential alcohol cessation treatment program appear to be at increased risk of post-treatment relapse [11].

In patients with AUD, improving sleep may support recovery and promote abstinence from alcohol [6]. Cognitive behavioral therapy has been shown to improve insomnia and alcohol-related outcomes in individuals with heavy drinking [12] and AUD [13]. Another non-pharmacological intervention known to improve sleep in non-clinical populations is physical activity. Moreover, Adapted Physical Activity (APA) provides several additional health benefits, including improvements in comorbidities, craving, and mental health, and appears to be a feasible treatment that is well received by patients with AUD [14], with high adherence rates reported in previous studies [15, 16]. However, unlike cognitive behavioral therapy, the impact of physical activity on sleep has not yet been tested in this specific population. This warrants further investigation.

The positive effects of physical activity on sleep in the general population are well established. Acute exercise—defined as less than one week of aerobic and/or anaerobic activity—improves TST, SE, and SWS, while reducing SOL, REM sleep, WASO, and stage 1 sleep, when performed at least three hours before bedtime [17]. These immediate benefits may motivate individuals to engage in regular physical activity to improve sleep [17]. Regular exercise—defined as at least one week of aerobic and/or anaerobic activity—is associated with improvements in TST, SE, SOL, and overall sleep quality, as well as a reduced need for sleep medication [18], particularly among individuals with pre-existing sleep complaints [17, 18]. Physical activity also contributes to circadian clock regulation by modulating melatonin secretion depending on the time of day, light exposure, exercise intensity, and duration [10]. In addition, regular physical activity decreases cortisol levels, which are correlated with changes in TST [19]. Although the relationships between sleep and alcohol use, and the independent benefits of physical activity on both, are well established, evidence remains limited regarding the effects of physical activity on sleep in individuals with AUD. However, recent literature suggests potential sleep-related benefits of physical activity in this population [20].

Patients with AUD may be receptive to APA because it may be perceived as less stigmatizing than other interventions [14, 21]. Patients may also perceive broad benefits of physical activity as an adjunctive treatment, extending beyond sleep to include improvements in alcohol consumption, craving, abstinence, mental health, physical condition, and quality of life [22, 23]. Despite this, patients with AUD and insomnia are less likely to engage in physical activity than those without insomnia (21% vs. 70%) [15].

This pilot randomized controlled trial aims to assess the feasibility and estimate the effects of a supervised adapted physical activity program primarily on sleep outcomes, and secondarily on physical and mental health parameters, in patients with AUD. The protocol of this study is presented below in accordance with the SPIRIT 2022 checklist guidelines.

## Methods

### Study design and objectives

This study is a feasibility randomized controlled trial (RCT) with two parallel groups of individuals with AUD. The allocation ratio is 3:1, with three participants assigned to the intervention group for every one participant in the control group. This pilot RCT follows the CONSORT guidelines for randomized trials. The clinical trial is registered under the number NCT06706349 (registered on 2 June 2024; last updated on 26 November 2024) and is sponsored by CHU Besançon, France. All data are collected at a single research site. Final approval from the relevant ethics committees was obtained in September 2024.

The primary objective of this study is to assess the feasibility of conducting an RCT exploring the impact of physical activity on sleep in patients with AUD. The secondary objective is to evaluate the effects of a supervised physical activity program on sleep, addiction severity, physical fitness, functional capacity, and mental health in this population.

### Study setting

**Fig. 1:**
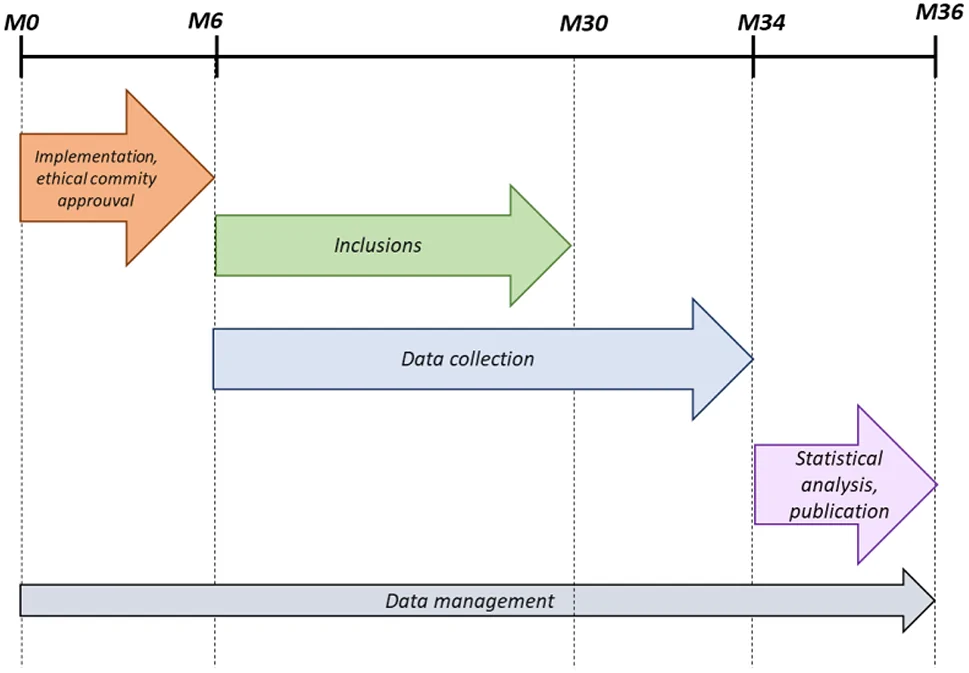
Flowchart of the study timeline. *M* = month

The study is conducted at the Day Hospital for Addictology (DAH) of the University Hospital of Besançon (CHU Besançon, France), an outpatient unit. Recruitment began in October 2024 and is expected to continue for 24 months, ending in October 2026. The total study duration is three years, with completion anticipated in April 2027, as illustrated in Fig. 1.

### Participants

#### Eligibility criteria

The study population includes men and women aged 18 to 65 years who are able to engage in Adapted Physical Activity (APA), have a diagnosis of alcohol use disorder (AUD) with ongoing alcohol consumption, and report sleep complaints. Participants must not be highly physically active and must not have any other substance use disorder, except for tobacco use. Specific inclusion and non-inclusion criteria are detailed in Table 1.

**Table 1:** Inclusion and non-inclusion criteria of APhysOH study

Inclusion criteria	Non-inclusion criteria
• Aged 18 to 65 years • AUD with ≥2 DSM-5 criteria • Sleep complaint with PSQI score > 5 • At least 6 days of excessive alcohol consumption in the past 4 weeks • Able to speak and understand French • Living within 30 minutes of the hospital • Stable pharmacological treatment (no changes in the past 4 weeks) • Affiliated with French social security • For women: menopausal for at least 24 months, surgically sterilized, or using effective contraception	• Absolute contraindication to APA according to HAS guidelines (e.g., decompensated heart failure) • Breath alcohol level above 0 on the day of consent • Alcohol consumption below inclusion threshold (less than 2 units per day or less than 10 per week) • Substance use disorder other than alcohol or nicotine • High physical activity level (GPAQ) • Withdrawal symptoms with CIWA score ≥ 9 • Acute psychiatric disorder requiring immediate hospitalization • History of delirium tremens • Anticipated poor compliance with study procedures (e.g., difficulty completing alcohol monitoring over 3 months) • Recent changes in psychotropic medication • Legal incapacity • Treatment with melatonin, beta-blockers, or disulfiram (Antabuse) • Participation in another study during an exclusion period • Pregnancy

APA Adapted Physical Activity; DSM-V Diagnostic and Statistical Manual of Mental Disorders, Fifth Edition; PSQI Pittsburgh Sleep Quality Index; HAS Haute Autorité de Santé; CIWA Clinical Institute Withdrawal Assessment for Alcohol; GPAQ Global Physical Activity Questionnaire

#### Recruitment procedures

Participants are recruited through multiple channels. Articles about the study are published in the local press. Information is also disseminated via the hospital website and social media, and posters and flyers are displayed in several hospital departments. As part of routine care activities, the DAH informs its partners (e.g., general practitioners, addiction support services) about the study. Finally, the study is presented at local meetings involving regional stakeholders in addictology.

#### Sample size

A total of 40 participants with AUD, ongoing alcohol consumption, and sleep complaints will be recruited, in accordance with recommendations for pilot and feasibility studies [24]. Participants will be randomized in a 3:1 ratio: 30 in the intervention group and 10 in the control group. This allocation balances the need for reliable estimates while accounting for limited recruitment capacity, in line with CONSORT 2010 guidelines [25].

The control group will serve as an internal reference to descriptively estimate (via 95% confidence intervals, without formal statistical testing) the magnitude of differences between groups. This approach provides a more relevant estimate of the potential effect of APA for designing a future superiority trial than relying solely on external data, and is not affected by unequal group sizes.

This sample size is sufficient to generate hypotheses for future trials. If the results are promising, the findings will support the design of a larger randomized trial evaluating the effectiveness of APA. The proposed sample size of 40 participants is justified, as 30 subjects in the intervention group is considered adequate to establish feasibility in quantitative studies [26], while a control group of 10 aligns with established rules of thumb for pilot trial control arms [27].

### Intervention

#### Supervised group (intervention)

All participants receive usual care (e.g., psychotherapy, and medication if stabilized and not contraindicated). Participants randomized to the supervised group will additionally receive a supervised APA program.

Prior to starting the APA program, a motivational interview is conducted with each participant by an APA instructor. The aim of this interview is to identify participants’ preferences, needs, capacities, and limitations. Based on this information, physical activity sessions are tailored to individual characteristics to enhance adherence.

The APA program was developed in accordance with national guidelines and scientific evidence regarding the effects of exercise on sleep, while also taking into account population-specific characteristics. It consists of a 12-week program including moderate- to high-intensity sessions combining aerobic and anaerobic exercise. Three group-based sessions per week are scheduled.

All sessions are supervised by an APA instructor, take place from 9:00 to 10:00 a.m., and include a maximum of six participants per session. All participants arrive at the same time. Using a progressive approach, participants are integrated into the group, and sessions are individually adapted according to functional capacity and therapeutic needs. Peer support and social interaction are actively encouraged.

The program components—frequency, intensity, time, type, and duration (FITT-D)—are detailed and justified with scientific references in Table 2.

**Table 2:** APA program design and rational

	APA program characteristics	Scientific background and justification
Frequency	3 one-hour sessions per week	· Sessions should be as regular as possible to enhance sleep [28]. More than three sessions per week may be too demanding for sedentary AUD patients. Longer sessions improve sleep (e.g., reduced SOL), but >60 min may be unsuitable [17].
Intensity	Moderate to high *6, 7, 8/10 Borg CR10*	· Moderate-to-high intensity (~75–80% VO2max) improves sleep [17].
Time	Morning *Before 12 p.m.*	· Although no consensus exists, systematic reviews suggest morning exercise enhances sleep benefits [29]. Additionally, morning physical activity reduces the risk of individuals arriving intoxicated, unfit for exercise.
Type of APA	Supervised group sessions *Aerobic + Anaerobic* *Adapted Pilates, step, boxing, cycling, walking/running, team sports*	· Supervision improves adherence [30]. No difference between aerobic/anaerobic for sleep outcomes [17]. Group formats improve adherence [31].
Duration	12 weeks	· A 12-week duration is commonly reported as sufficient to observe sleep improvements [28].

APA Adapted Physical Activity, SOL Sleep Onset Latency, BorgCR10 Borg Category Ratio 10 scale, SWS Slow Wave Sleep

Concerning the type of program, in general:

- **Mondays** focus on individual-type activities with full-body workouts incorporating cardiovascular, strength, and coordination components (e.g., boxing, step training).
- **Tuesdays** are dedicated to mind–body and muscular strengthening activities (e.g., Pilates, yoga).
- **Fridays** focus on outdoor activities or adapted team sports (e.g., hiking, basketball, rugby).

Overall, the program combines aerobic and anaerobic exercise while promoting social engagement and the development of motor and psychological skills.

#### Autonomous group (control)

In this study, the control condition—referred to as the “autonomous group”—is designed as a minimal-level usual care intervention. Participants already engaged in addiction treatment will continue their usual care, while those not yet in care will receive standard treatment initiation.

All participants in the control group will also receive standardized verbal and written recommendations regarding physical activity. This design enhances external validity by reflecting routine clinical practice and maintains ethical standards by ensuring that participants are not deprived of basic health advice, while preserving the integrity of an active control condition through standardized messaging [32].

### Assignment of interventions

#### Sequence generation and allocation concealment

Randomization is performed at inclusion using CleanWeb®, based on a pre-established allocation sequence generated by a data manager. The allocation is concealed from the investigators and assigns participants randomly in a 1:3 control-to-intervention ratio. Investigators are responsible for enrolling participants and assigning them to groups.

#### Blinding

After randomization, neurologists and nurses from the sleep unit responsible for sleep assessments are blinded to group allocation. Investigators, who inform participants of their group assignment, and the APA instructor conducting the sessions are not blinded. Participants themselves cannot be blinded due to the nature of the intervention (physical activity).

### Outcomes

#### Primary outcome

The primary outcome is to assess the feasibility of the APhysOH pilot RCT. This includes evaluating whether the supervised APA program meets a demand and generates interest among individuals with AUD, as well as assessing recruitment capacity for both study arms. Acceptability of randomization, adherence, and attendance over the 12-week program will also be evaluated.

#### Measurements

Feasibility will be assessed using multiple indicators of compliance and engagement. These include:

1. The proportion of individuals enrolled after attending the initial information session;
2. The proportion of enrolled participants who complete the full study protocol;
3. Completion rates by group;
4. In the intervention group, the proportion of participants attending at least two-thirds of APA sessions.

Reasons for non-inclusion will be documented to identify potential recruitment barriers. Sociodemographic characteristics will be collected to describe the study population. Acceptability of randomization will be inferred from post-inclusion withdrawal rates.

Participant engagement will be assessed using self-reported physical activity diaries. In the intervention group, attendance at APA sessions—including dates, presence, and reasons for absence—will be recorded to evaluate adherence and identify barriers to sustained participation.

Feasibility will also be evaluated through completion rates of questionnaires, physical tests, and activity diaries.

Finally, the benchmarks determining whether the study is feasible or not are as follow:

- At least 75% of the planned sample size will be recruited within the initially planned recruitment period.
- At least 50% of participants will be adherent to the assigned intervention, defined as completing more than 50% of the planned APA sessions (intervention group).
- At least 50% of participants will successfully complete the sleep analysis assessments.
- At least 50% of participants will complete the follow-up period.

#### Secondary outcomes

##### Sleep

Sleep outcomes aim to quantify the effects of the supervised APA program on both objective and subjective sleep parameters in individuals with AUD and sleep complaints. Another objective is to identify the most sensitive psychometric sleep scales for detecting changes associated with the intervention.

###### Measurements

Objective sleep is assessed before (T1) and after (T3) the intervention using validated tools and circadian rhythm analyses. These include actigraphy [33] and measurement of 6-sulfatoxymelatonin concentrations [34].

Participants wear an actimeter (MotionWatch 8, CamNtech, class I CE medical device) for 14 consecutive nights at T1 and T3. This device measures: Sleep duration (SD); Sleep efficiency (SE); Sleep onset latency (SOL); Wake after sleep onset (WASO); Fragmentation index (FI); Non-parametric circadian rhythm analysis (NPCRA) markers. NPCRA markers include:

- Onset time and mean activity during the least active 5 hours (L5) and most active 10 hours (M10)
- Relative amplitude (RA), defined as the normalized difference between M10 and L5
- Inter-daily stability (IS), reflecting day-to-day rhythm regularity
- Intra-daily variability (IV), reflecting within-day rhythm fragmentation

Following each 14-day actigraphy period (T1 and T3), participants collect eight urine samples over 24 hours (every 3 hours). These samples are used to determine the acrophase of 6-sulfatoxymelatonin, a standard biomarker of circadian rhythms.

These objective measures were selected because they allow home-based assessment, which is more suitable for patients with AUD.

Subjective sleep outcomes are assessed before (T1), during (T2), and after (T3) the intervention using sleep diaries and validated self-administered questionnaires. The main instruments include:

- Pittsburgh Sleep Quality Index (PSQI) for perceived sleep quality [35]
- Insomnia Severity Index (ISI) for insomnia severity [36]
- Horne and Östberg questionnaire for chronotype [37]

These questionnaires are easy to complete, validated and widely used in the literature in non-clinical population and in patients with AUD [38]. Participants also complete a 14-day sleep diary at T1 and T3 while wearing the actimeter. This diary provides self-reported measures of SD, SE, SOL, WASO, and number of awakenings, allowing assessment of perceived sleep quality [39].

##### Addiction, physical, and mental health

The second set of outcomes aims to evaluate the effects of the supervised APA program on: Addiction severity; Physical fitness; Functional capacity and Mental health.

###### Measurements

The instruments used to assess these outcomes are listed in Table 3, with detailed descriptions provided in the supplementary materials.

### Data collection

#### Participant timeline

**Fig. 2:**
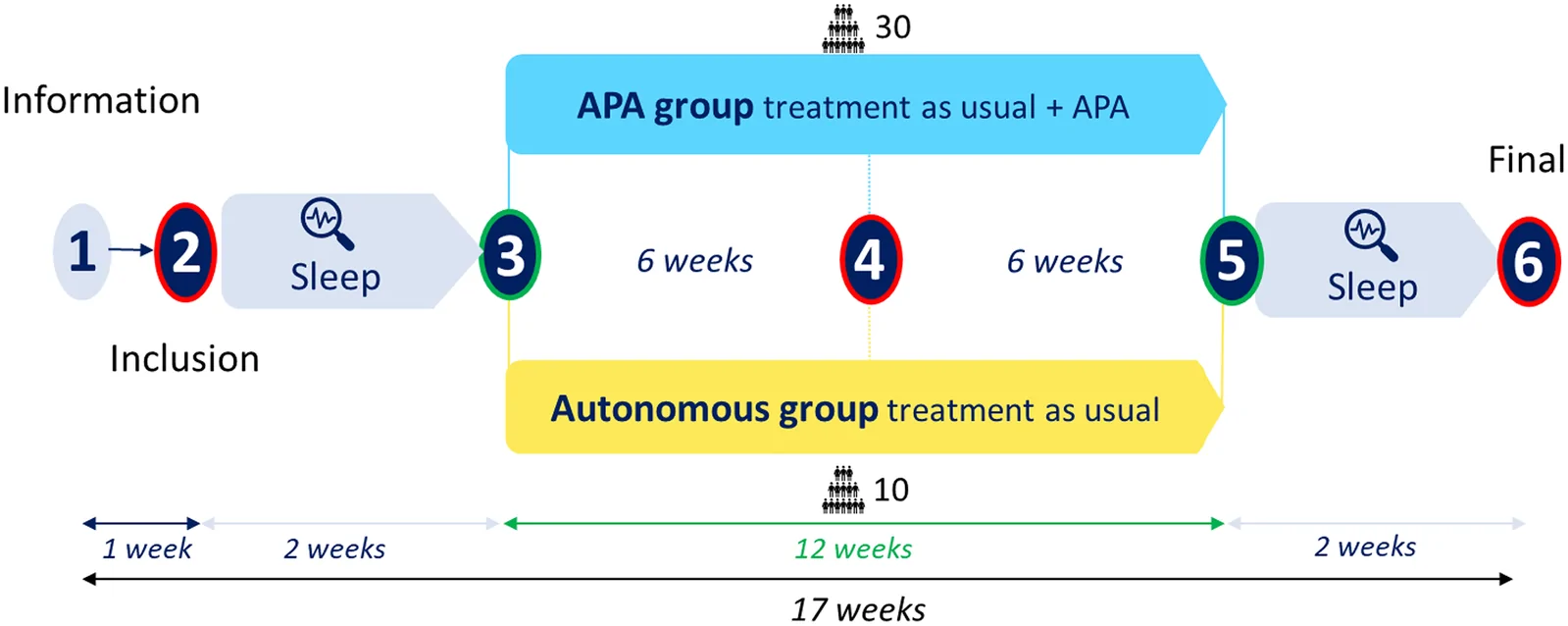
Participants timeline - APhysOH study. *n* = number; *T* = Time; V = visit

The study lasts 17 weeks, from the information visit (T0) to the final visit (T3). A minimum of one week is required between the information visit and inclusion (T1). Two additional weeks are dedicated to sleep assessment before and after the 12-week intervention. The study timeline is presented in Fig. 2.

- **Information visit (T0):** Interested participants receive detailed information about the study protocol. Investigators answer questions, collect sociodemographic data, and provide the information sheet.
- **Inclusion visit (T1):** At least 7 days after T0, participants return to the DAH for their second visit (V2). A psychiatrist specialized in addiction assesses eligibility. If all criteria are met and breath alcohol concentration is 0 mg/L, written informed consent is obtained. Randomization is then performed to assign participants to either the intervention group (supervised) or the control group (autonomous).Participants complete baseline assessments, including questionnaires, standardized tests, and biological sampling related to addiction, physical activity, mental health, and sleep. A sleep-focused medical consultation is conducted by a neurologist from the Sleep Exploration Unit (SEW). Participants are then provided with sleep monitoring devices and record their sleep at home for approximately 14 days. After returning the sleep monitoring devices, participants either begin the supervised APA program or continue usual care (V3).
- **Intermediate visit (T2):** Midway through the intervention (visit 4), participants complete follow-up questionnaires on addiction, sleep, mental health, and physical activity.
- **Final visit (T3):** At the end of the intervention, participants undergo a second sleep consultation (visit 5) and receive sleep monitoring devices again. After another 14-day home monitoring period, they return the devices and complete final assessments (visit 6), including all measures performed at baseline.

#### Data collection procedures

Data are initially recorded on paper case report forms (CRFs) and subsequently transcribed into electronic CRFs (eCRFs) using CleanWeb®. As shown in Table 3, data are collected using a range of validated tools assessing all study outcomes at multiple time points (before, during, and after the intervention). Data collection involves several professionals, including investigators, nurses, APA instructors, neurologists, and psychiatrists.

**Table 3:**
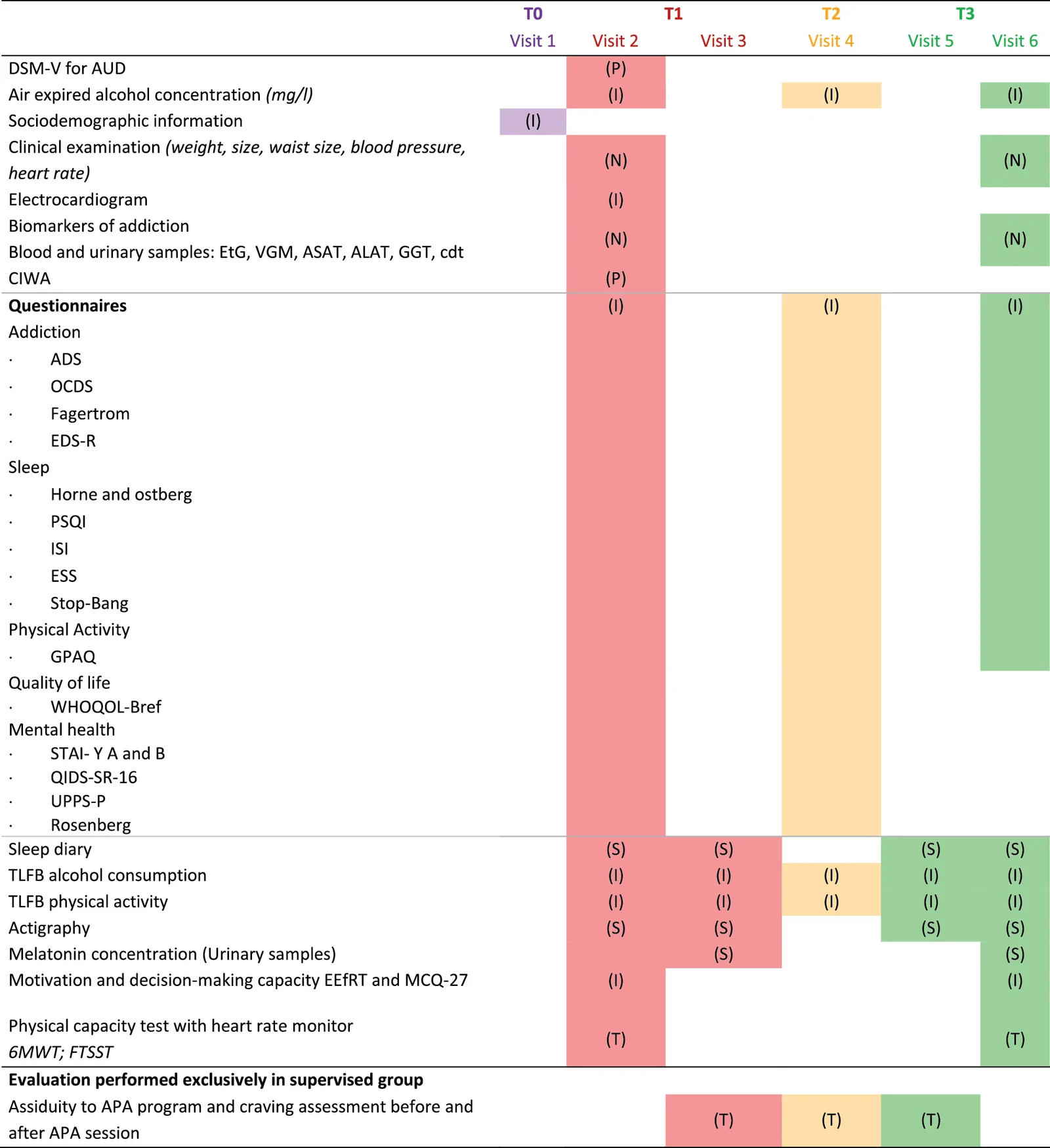
Data collection procedure and timeline

(P) Psychiatrist; (I) Investigators; (N) DAH Nurses; (T) Teachers in APA; (S) Neurologists or sleep ward nurses; APA Adapted Physical Activity; DSM-V Diagnostic and Statistical Manual of Mental Disorders, Fifth Edition; CIWA Clinical Institute Withdrawal Assessment for Alcohol; ADS Alcohol Dependence Scale; OCDS Obsessive Compulsive Drinking Scale; EDS-R Exercise Dependence Scale; PSQI Pittsburgh Sleep Quality Index; ISI Insomnia Severity Index; ESS Epworth Sleepiness Scale; GPAQ Global Physical Activity Questionnaire; WHOQOL World Health Organisation Quality Of Life; STAI-Y Stait Trait Anxiety Inventory; QIDS-Sr-16 Quick Inventory of Depressive Symptomatology; UPPS-P Impulsive Behaviour Scale; TLFB Time Line Follow Back; EEfRT Effort Expenditure for Reward Task; MCQ-27 Monetary Choice Questionnaire; 6MWT 6-minute walking test; FTSST Five Times Sit to Stand Test

### Data management

Data quality control includes verification of 20% of CRFs selected at random by an independent operator throughout the study to ensure transcription accuracy.

All data are stored on secure, dedicated servers with restricted access. After database lock, only the data manager will have access to the finalized dataset. Other authorized personnel involved in data analysis will be granted read-only access.

#### Monitoring

The study will be monitored by a Clinical Research Associate appointed by the sponsor, independently of the investigators, in accordance with CHU Besançon procedures and applicable regulations. Monitoring visits will include review of investigator files and informed consent forms.

No independent Data Monitoring Committee has been established due to the single-center design and the low-risk nature of the study.

The sponsor and principal investigator reserve the right to suspend or terminate the trial prematurely for medical or administrative reasons, upon mutual agreement. Reasons for early termination will be documented and reported to the relevant authorities. Participants may withdraw their consent at any time without consequences.

Additional stopping criteria include technical issues preventing study continuation, participant inability to complete study procedures, death, loss to follow-up, or failure to meet predefined feasibility thresholds (e.g., insufficient recruitment or excessive dropout rates).

#### Harms

In accordance with French Jardé law (L.1123–10), all adverse events occurring after informed consent must be reported in compliance with regulatory requirements. This includes events related to medication, medical devices, or care procedures. Investigators will report adverse events through the ENNOV system, specifying trial participation. Any new safety information must be promptly communicated to the local vigilance unit.

#### Auditing

The study may be audited by a Clinical Research Associate mandated by the sponsor, independently of the investigators, in accordance with CHU Besançon procedures and applicable regulations. Audits will include review of investigator files and consent documentation.

### Statistical analysis

Statistical analyses will be primarily descriptive. The study aims to describe feasibility outcomes and to estimate differences between the APA and autonomous groups for secondary outcomes. Measures of central tendency and dispersion will be reported. For quantitative variables, these include the mean, median, first and third quartiles, minimum and maximum values, range, variance, standard deviation, and coefficient of variation. For qualitative variables, absolute and relative frequencies will be presented. Data will also be displayed graphically using histograms, pie charts, and bar charts.

As a feasibility study, this trial is not powered to detect treatment effects. Differences between groups will be reported descriptively with 95% confidence intervals, without formal hypothesis testing. These preliminary estimates, based on a small sample, are intended solely to inform the design of future adequately powered trials and should not be interpreted as definitive effect estimates.

Adherence to the intervention and follow-up will be described over time.

## Discussion

### Limitations

This pilot randomized controlled trial evaluates the feasibility of assessing the effects of physical activity on sleep in individuals with AUD. A major challenge is that this population may experience difficulties adhering to care. Indeed, studies report that only 21% of patients with AUD consistently adhere to prescribed medications [40]. Although adherence to supervised physical activity programs has been reported to range from 70% to 78% [15, 16], it may drop to 21% among patients with both AUD and insomnia [15]. Therefore, adherence and attendance represent key feasibility concerns, highlighting the importance of feasibility outcomes to inform future RCTs.

Another limitation is the small sample size, which requires confirmation of the effects of physical activity on sleep in larger studies. In addition, although polysomnography would provide a more detailed assessment of sleep architecture, its requirement for overnight hospitalization may reduce acceptability in this population.

Furthermore, changes in alcohol consumption during the intervention could confound the effects of physical activity on sleep. However, sleep disturbances associated with chronic alcohol use are known to persist for months or even years after cessation [4, 6]. Future adequately powered studies should investigate whether changes in alcohol use moderate the effects of physical activity on sleep outcomes.

Finally, substantial interindividual variability is expected. Future research may benefit from incorporating n-of-1 designs or complementary qualitative approaches to better characterize within-person effects of physical activity on sleep and related health outcomes [41].

## Conclusion

This pilot RCT addresses a gap in the literature by evaluating an intervention targeting both sleep and addiction-related outcomes. The findings will inform the design of future randomized controlled trials by providing insights into feasibility, protocol implementation, assessment strategies, and intervention delivery.

In addition, this study will generate preliminary data on the potential effects of physical activity on sleep, substance use, physical health, and mental well-being in individuals with AUD. Future studies should build on these findings to design adequately powered trials, explore the effects of physical activity across different stages of AUD, and refine exercise prescriptions by comparing different intervention modalities.

## Electronic supplementary material

Below is the link to the electronic supplementary material.

Supplementary Material 1

## Data Availability

Access to the final trial dataset will be restricted to the Sponsor and authorized personnel at CHU Besançon, given the presence of sensitive individual health information of patients. External parties interested in data access may contact the corresponding author to discuss data sharing possibility in accordance with ethical and regulatory requirements.

## Abbreviations

ADS: Alcohol Dependence Scale
APA: Adapted Physical Activity
AUD: Alcohol Use Disorder
CIWA: Clinical Institute Withdrawal Assessment for Alcohol
DSM-V: Diagnostic and Statistical Manual of Mental Disorders, Fifth edition
EDS-R: Exercise Dependence Scale-Revised
ESS: Epworth Sleepiness Scale
FTSST: Five Times Sit to Stand Test
FI: Fragmentation Index
GPAQ: Global Physical Activity Questionnaire
IS: Inter-daily Stability
IV: Inter-daily Variability
ISI: Insomnia Severity Index
L5: Least active 5 hours
M10: Most active 10 hours
NPCRA: Non-Parametric Circadian Rhythm Analysis
OCDS: Obsessive Compulsive Drinking Scale
PSQI: Pittsburgh Sleep Quality Index
QIDS-Sr-16: Quick Inventory of Depressive Symptomatology
RA: Relative Amplitude
RCT: Randomized Controlled Trial
SD: Sleep Duration
SE: Sleep Efficiency
SOL: Sleep Onset Latency
STAI-Y: Stait Trait Anxiety Inventory
TLFB: Time Line Follow Back
UPPS-P: Impulsive Behavior Scale
WASO: Wake After Sleep Onset
WHOQOL: World Health Organization Quality of Life
6MWT: 6-minute walking test

## Declarations

### Protocol amendments

This is a single-center study with a clear responsibility matrix defining communication roles. The Principal Investigator (PI) requests the modification submission, which is prepared and followed up by the project manager. After positive feedback from the Ethics Committee (CPP) is received by the Research Department (DRCI), the PI, project manager, and all relevant parties are promptly informed.

### Confidentiality

The Sponsor is the data controller and commits to collecting only data strictly necessary for the research objectives. Data are pseudonymized using a unique code, excluding direct identifiers. Only the investigator and authorized staff can link codes to participant identities. A responsibility policy ensures data security and confidentiality, compliant with applicable regulations (including MR001 and GDPR). Participants are informed about data use, their rights, and data retention periods. Data collection sources are the participants themselves, their legal representatives, and involved professionals.
