# Supplementary material for: Physical activity and sleep disturbances in individuals with alcohol use disorder: protocol of the APhysOH pilot study for a randomized controlled trial
Source: Addict Sci Clin Pract. 2026 Jun 13;21:69. doi: 10.1186/s13722-026-00683-1 (PMC13628940; doi:10.1186/s13722-026-00683-1)
Supplement: Supplementary file 1 [file 13722_2026_683_MOESM1_ESM.docx]

# Supplementary material

## Measuring tools assessing secondary outcomes

#### **Physical activity**

**A clinical examination collects anthropometric and cardiovascular markers, including height, weight, waist circumference, blood pressure, and heart rate. During physical tests, heart rate is monitored using a CL800 Chest Strap Heart Rate Monitor.**

**The 6-Minute Walk Test (6MWT) evaluates cardiovascular and functional capacity. Participants walk as far as possible in 6 minutes without running. Distance and heart rate are recorded at baseline, minute 3, minute 6, and one minute post-test. This test is validated for assessing functional capacity in AUD inpatients**^4^**. The Five Times Sit-to-Stand Test (FTSST) also assesses functional capacity. Participants stand and sit five times as quickly as possible. Time and heart rate are recorded at baseline, immediately after the test, and one minute post-test**^5^**.**

**Subjective physical activity is assessed with the Global Physical Activity Questionnaire (GPAQ), developed by World Health Organization (WHO)**^6^**. It captures activity across work, transport, and leisure domains, with scores expressed in** Metabolic Equivalent Tasks’s (**MET)-minutes/week. Participants are classified as having low, moderate, or high activity levels. An exercise log is provided to both groups, documenting date, activity type, perceived intensity (rated on the Borg CR10 scale, 1–10), and session duration. Effort perception is measured using the Borg Rating of Perceived Exertion (RPE) scale. This study uses the validated CR10 version, ranging from 1 (minimal effort) to 10 (maximal effort)** ^7^**.**

#### Addiction analysis

Urinary ethyl glucuronide (EtG) is a direct biomarker of ethanol, detectable for up to 72 hours after the last alcohol intake. A small proportion of ethanol escapes hepatic oxidation and is excreted in the urine as EtG following conjugation with glucuronic acid. Its half-life after complete ethanol elimination is approximately 2 to 3 hours, which accounts for its detectability up to 72 hours. Studies stratifying consumption levels show that sensitivity increases with the amount consumed, ranging from 30.3% for 1 to 4 units per month to 96.3% for 4 units per day. This makes EtG a reliable marker of excessive alcohol consumption. Traditional indirect biomarkers of alcohol use include carbohydrate-deficient transferrin (CDT), aspartate aminotransferase (AST), alanine aminotransferase (ALT), gamma-glutamyl transpeptidase (GGT), and mean corpuscular volume (MCV).

The DSM-5 section on Alcohol Use Disorder serves as the standard diagnostic tool for identifying problematic alcohol use during investigator-led interviews.

Self-report assessments include several validated tools. The Fagerström Test for Nicotine Dependence is widely used in medical consultations and pharmacies to evaluate the need for nicotine replacement therapy. Although tobacco use is not an exclusion criterion, this test helps control for its potential confounding effects. The Alcohol Dependence Scale (ADS-Fr) is a 25-item questionnaire that provides a quantitative measure of alcohol dependence; a score greater than 9 is highly predictive of an alcohol use disorder. The Timeline Follow Back (TLFB) method allows participants to report their alcohol consumption using a standard unit equivalent to 10 grams of ethanol. The Exercise Dependence Scale–Revised (EDS-R) consists of 21 items that evaluate seven dimensions of addiction: withdrawal, tolerance, loss of control, reduction of other activities, time spent, intention, and continuation despite consequences. Each dimension is considered positive if the total score of its three associated items exceeds 15, and a diagnosis of exercise addiction is made when at least three dimensions are present.

Craving is assessed using a Visual Analog Scale (VAS) with a Likert scale, where participants rate their desire to drink from 0 (none) to 10 (extremely strong and uncontrollable). The Obsessive Compulsive Drinking Scale (OCDS), validated in French, consists of 14 items and assesses the cognitive components of alcohol craving, as well as the efficacy of interventions over 12 weeks. The Clinical Institute Withdrawal Assessment for Alcohol–Revised (CIWA-Ar) is a 10-item clinician-administered scale used to assess the presence and severity of alcohol withdrawal syndrome and to guide benzodiazepine treatment. CIWA-Ar scores are interpreted as follows: 0 indicates no withdrawal, 1 to 8 indicates mild withdrawal, 9 to 15 indicates moderate withdrawal, and scores above 15 indicate severe withdrawal.

#### Mental Health analysis

Quality of life was assessed using the WHOQOL-Bref, a brief 26-item self-report questionnaire developed by the World Health Organization to evaluate general quality of life.

Mental health was evaluated through several self-report instruments. The State-Trait Anxiety Inventory (STAI) Form Y consists of two 20-item subscales measuring trait anxiety (personality characteristics) and state anxiety (response to situational stressors), with scores ranging from 20 to 80. The Rosenberg Self-Esteem Scale includes 10 items with four response options ranging from strong disagreement (1 point) to strong agreement (4 points); scores are interpreted as low self-esteem (10–16 points), moderate self-esteem (17–33 points), and high self-esteem (34–40 points). The UPPS-P Impulsive Behavior Scale is a 20-item questionnaire assessing five dimensions of trait impulsivity: positive urgency, negative urgency, lack of premeditation, lack of perseverance, and sensation seeking. Evaluating personality profiles is important as impulsivity traits may predict treatment adherence or dropout. The Quick Inventory of Depressive Symptomatology–Self Report (QIDS-SR-16) is a 16-item questionnaire measuring the nine DSM-5 symptoms of a major depressive episode, with scores ranging from 0 to 27.

Decision-making and motivational capacities were assessed using two tasks. Delay discounting was evaluated with the Monetary Choice Questionnaire (MCQ-27), a 27-item measure presenting intertemporal choices between smaller immediate and larger delayed rewards, with amounts pre-set to represent different reward discounting rates. Motivation was assessed using the Effort Expenditure for Reward Task (EEfRT) adapted for a cycle ergometer, which measures the effort a subject is willing to exert for a given reward. In each trial, the participant chooses between an easy and a hard task based on the probability of obtaining the associated reward. The tasks differ by pedaling resistance, which must be maintained for a set time to fill a gauge. Resistance levels are calibrated initially based on the participant’s physical capacity, with the easy task requiring low resistance and the hard task requiring high resistance.

## Bibliography

1.	Dietch JR, Taylor DJ. Evaluation of the Consensus Sleep Diary in a community sample: comparison with single-channel electroencephalography, actigraphy, and retrospective questionnaire. *J Clin Sleep Med JCSM Off Publ Am Acad Sleep Med*. 2021;17(7):1389-1399. doi:10.5664/jcsm.9200

2.	Buysse DJ, Reynolds CF, Monk TH, Berman SR, Kupfer DJ. The Pittsburgh Sleep Quality Index: a new instrument for psychiatric practice and research. *Psychiatry Res*. 1989;28(2):193-213. doi:10.1016/0165-1781(89)90047-4

3.	Bastien CH, Vallières A, Morin CM. Validation of the Insomnia Severity Index as an outcome measure for insomnia research. *Sleep Med*. 2001;2(4):297-307. doi:10.1016/s1389-9457(00)00065-4

4.	Vancampfort D, Vandael H, Hallgren M, Van Damme T. Test-retest reliability and correlates of the 6-min walk test in people with alcohol use disorders. *Physiother Res Int J Res Clin Phys Ther*. 2021;26(3):e1900. doi:10.1002/pri.1900

5.	Muñoz-Bermejo L, Adsuar JC, Mendoza-Muñoz M, et al. Test-Retest Reliability of Five Times Sit to Stand Test (FTSST) in Adults: A Systematic Review and Meta-Analysis. *Biology*. 2021;10(6):510. doi:10.3390/biology10060510

6.	Bull FC, Maslin TS, Armstrong T. Global physical activity questionnaire (GPAQ): nine country reliability and validity study. *J Phys Act Health*. 2009;6(6):790-804. doi:10.1123/jpah.6.6.790

7.	Noble BJ, Borg GA, Jacobs I, Ceci R, Kaiser P. A category-ratio perceived exertion scale: relationship to blood and muscle lactates and heart rate. *Med Sci Sports Exerc*. 1983;15(6):523-528.
